# Stereoelectroencephalography for drug resistant epilepsy: precision and complications in stepwise improvement of frameless implantation

**DOI:** 10.1007/s00701-025-06489-5

**Published:** 2025-03-17

**Authors:** Tatjana Liakina, Andreas Bartley, Louise Carstam, Bertil Rydenhag, Daniel Nilsson

**Affiliations:** 1 Department of Clinical Neuroscience, Institute of Neuroscience and Physiology, Sahlgrenska Academy, University of Gothenburg, Gothenburg, Sweden; 2 Department of Clinical Neurophysiology, Sahlgrenska University Hospital, Member of the ERN EpiCARE, Gothenburg, Sweden; 3 Department of Neurosurgery, Sahlgrenska University Hospital, Member of the ERN EpiCARE, Blå Stråket 5, 3rd Floor, Gothenburg, 413 45 Sweden

**Keywords:** Epilepsy surgery, Stereoelectroencephalography, Drug resistant epilepsy, Neuronavigation, Robotics, Precision

## Abstract

**Purpose:**

Stereoelectroencephalography (SEEG) is the standard for invasive investigations in epilepsy surgery. Our aim was to investigate if similar precision and low complication rate can be achieved with optimized frameless navigation as with frame-based or dedicated stereotactic SEEG robot.

**Methods:**

We compared five different implantation techniques assessing entry, target errors and complications in 53 SEEGs from 50 patients: Group 1 – surface registration and Vertek probe, Group 2 – rigid registration with conventional CT and Vertek probe, Group 3 – rigid registration and Vertek probe, Group 4 – rigid registration and Autoguide, Group 5 – rigid, sterile registration and Autoguide. Analysis was done using random effects linear modelling to calculate improvement in percent using Group 1 as a reference, p < 0.001 was considered significant.

**Results:**

Mean patient age at implantation was 23 years (range 4–46 years) and mean number of implanted electrodes per patient were 11 (range 3–15). Accuracy data was available for 36 SEEG implantations (419 electrodes). The median entry/target errors were (mm): Group 1:4.6/4.3; Group 2:1.8/2.3; Group 3:0.9/1.5; Group 4:1.1/1.2; Group 5:0/0.7. Improvement of accuracy for entry error was 38% for Group 2 (p = 0.004), 47% for Group 3 (p < 0.001), 50% for Group 4 (p < 0.001), and 72% for Group 5 (p < 0.001). Improvement of accuracy for target error was 17% for Group 2 (p = 0.17), 22% for Group 3 (p < 0.001), 35% for Group 4 (p < 0.001), and 51% for Group 5 (p < 0.001). Complications (hemorrhage, edema, headache) occurred in 7/53 SEEGs, none of these led to permanent deficit. 40/53 investigations resulted in an epilepsy surgery procedure.

**Conclusion:**

High precision and low complication rate in SEEG implantation can be achieved with frameless navigation using rigid, sterile registration.

## Introduction

Epilepsy surgery is a safe and effective treatment to achieve seizure control in patients suffering from medically refractory focal epilepsy[4, 12]. In many patients, surgical treatment can be offered exclusively based on results of non-invasive investigations. However, non-invasive investigations sometimes give equivocal or conflicting results. In these cases, invasive recording of brain cortical electrical activity is used to define a volume of cerebral cortex that needs to be resected to render the patient seizure-free or to significantly decrease seizure frequency.

Stereoelectroencephalography (SEEG) as a concept of introducing depth electrodes into the brain with the aim to record direct cortical activity in epilepsy patients was developed by Bancaud and Talairach in the 1960s [2, 3]. During the last decades, the technique has been adopted worldwide as a safe and efficient way to investigate focal epilepsy refractory to medical treatment [7, 20, 24, 29, 30, 36, 42, 44, 48].

As SEEG has become the new standard for invasive investigation in epilepsy surgery, there has been a growing interest in improving accuracy and workflow of this procedure. To perform SEEG safely and efficiently, high accuracy is a prerequisite, both to prevent haemorrhage and to hit deep, small targets in the brain. As the goal of SEEG is to map the anatomoelectroclinical network of the seizure start and propagation, SEEG routinely targets many deep and complex regions, such as the amygdala, hippocampus, insula (often with 2–5 electrodes) and deep medial/basal cortex. The planned trajectories frequently traverse arachnoid layers and ependyma.

Currently, three methods are used for placement of SEEG electrodes: stereotactic frame-based, frameless neuronavigation and robot assisted frameless neuronavigation. Stereotactic frame-based implantations, although considered gold standard in terms of accuracy, are time consuming and sub-optimal for implantations in young children due to a required minimal bone thickness for placement of the frame [5, 29, 33, 39]. Frameless neuronavigation techniques, although ubiquitous in the neurosurgical departments worldwide, have so far resulted in lower implantation precision than frame-based techniques [13, 34, 37, 40, 41]. Recent advances of robotic assisted neurosurgery seem to offer implantation precision comparable with frame-based techniques [1, 8, 10, 14, 15, 26–28, 32, 40, 41, 43, 48]. On the downside, this method comes with significant cost for purchase and maintenance of the robotic device and training of the personnel.

In this paper we report a single-centre experience with different frameless implantation methods, reporting precision of implantation and complications. Our aim was to investigate if similar precision and low complication rate can be achieved with optimized frameless navigation as with frame-based or dedicated stereotactic robot SEEG.

## Methods

### Study population

Between 2013–2023, 53 SEEG implantations were performed in 50 consecutively included patients with refractory focal epilepsy at Sahlgrenska University Hospital, referral center for advanced epilepsy surgery and one of six major neurosurgical sites in Sweden. The natural continuous efforts to improve the SEEG implantation procedure has allowed for a retrospective comparison of five different implantation techniques assessing accuracy and complications. The study cohort is described in Table 1.

**Table 1:** Descriptive data of the 53 stereoelectroencephalographies (SEEG) carried out in 50 patients

		SEEG implantations in Gothenburg between 2013—2023		
		Children (*n* = 16)	Adults (*n* = 37)	All (*n* = 53)
Male / Female		9 / 7	18 / 19	**27 / 26**
Age at implantation, years	Mean (SD)	11 (5)	29 (9)	**23 (11)**
	Median	11	26	**23**
	[Min – Max]	[4–17]	[18–46]	**[4–46]**
Disease duration, years	Mean (SD)	5 (3)	15 (9)	**12 (9)**
	Median	5	13	**10**
	[Min – Max]	[1–12]	[3–43]	**[1–43]**
Follow up, years	Mean (SD)	6 (3)	5 (3)	**6 (3)**
	Median	6	5	**5**
	[Min – Max]	[1–11]	[1–11]	**[1–11]**
MRI positive, n (% of respective age group)		10 (62.5%)	16 (43.2%)	**26 (49.1%)**
No of implanted electrodes per patient, n	Mean (SD)	11 (3)	11 (2)	**11 (2.5)**
	Median	11	11	**11**
	[Min – Max]	[3–15]	[3–14]	**[3–15]**
Side of implantation, n (% of age group)	Left	10 (62.5%)	18 (48.7%)	**28 (52.8%)**
	Right	4 (25.0%)	16 (43.2%)	**20 (37.8%)**
	Bilateral asymmetric	2 (12.5%)	3 (8.1%)	**5 (9.4%)**
Monitoring duration, days	Mean (SD)	7 (3)	9 (3)	**8 (3)**
	Median	7	8	**7**
	[Min – Max]	[2–14]	[4–17]	**[2–17]**

### SEEG implantation procedure

#### Implantation planning

All implantation protocols utilised volumetric T1-weighted MRI for electrode trajectory planning, based on pre-invasive clinical, electrophysiological, neuropsychological, and multimodal imaging data to formulate hypothesis about seizure onset and propagation. T1 weighted contrasted MRI and CT-angiography were used for visualising the vascular structures. CT angiography was used as a reference modality for coregistration of any other volumetric or functional imaging due to decreased spatial distortion compared to MRI [31]. Coregistration and final planning was performed on a Stealth Station System (Medtronic Inc., USA). Versions S7 and S8 were used for patients between 2013 – 2018 and 2018 – 2023 respectively. A safety margin of 4 mm was used for the surface vessels and 2 mm for the deep vessels.

#### Evolution of registration technique

The first 18 patients were implanted using surface registration, which consists of fixing the patient’s head in the Mayfield or Sugita clamp and placing a probe on the anatomical landmarks identifiable both on the image coordinate space (CT-angiography) and the skin surface of the patients face and head, which are subsequently used for registration of operating and image coordinate spaces.

In 2017, the technique was modified to rigid-point registration, which consists of attaching radiopaque fiducials to the skull bone, then performing conventional head CT, which is pre-processed to identify the implanted fiducials and registered to the CT-angiography. With the patient’s head fixed in the Sugita head clamp, a probe is used to identify the fiducials, which are then used to align operating coordinate space and image coordinate space. The sterile preparation of the operating space was performed after neuronavigation alignment. From 2019 intraoperative O-arm (Medtronic Inc., USA) CT was used for the registration instead of a conventional CT.

The latest modification, introduced in 2022, consists of sterile O-arm CT acquisition and neuronavigation alignment performed after sterile preparation of the operating space. This reduces the risk of displacement of the head or neuronavigation antenna by the draping or by changing the antenna from non-sterile to sterile. Bone fiducials are identified in the operating field by the probe to confirm accuracy of registration before the start of the procedure.

#### Evolution of trajectory alignment technique

Until 2019, Vertek probe (Medtronic Inc., USA), which consists of placement of the probe on the patient´s skin followed by manual angle and position adjustment until alignment between the planned trajectory and the guiding tube is achieved using visual real time feedback on the navigation screen, was used for trajectory alignment.

Since 2020, Stealth Autoguide (Medtronic Inc., USA) system is used for semi-automated alignment procedure, which consists of initial manual placement of the system in line with approximate trajectory, which then automatically adjusts the angle and position to align the guiding tube with the planned trajectory. The precision of the alignment is given in millimetres in real time on the navigation screen.

Subsequent implantation procedure consists of pre-drilling the bone to 2–3 mm with a 2 mm diamond drill to prevent twist drill sliding on the outer surface of the bone. The final burr hole is made with the Dixi system (Dixi Medical) 2.3 mm twist drill, and a guiding screw (Dixi Medical) is placed. The distance to the target is measured using the navigation system, and a 0.8 mm Dixi Microdeep (Dixi Medical) electrode is inserted. Procedure is repeated until all planned electrodes are in place. Post-implantation CT is performed directly after procedure for localisation of electrode contacts using conventional CT before 2019 and intraoperative O-arm CT for later implantations.

### Implantation precision estimations

The data on the implantation precision was prospectively collected since 2017. For earlier implantations, data was retrospectively retrieved if available.

Accuracy measurements were available for 36 implantations (419 electrodes). 17 implantations excluded: no implantation accuracy data was available for the first 10 implantations (2013 – 2014), for another 5 implantations (2015 – 2020) planned trajectory files were unretrievable, 2 implantations (2021–2023) were excluded due to poor precision estimation related to material failure: navigation antenna detachment (1) or pressure on the navigation antenna support during operation (1). Further 6 electrodes in 5 implantations were excluded: no accuracy measurements were available for 2 electrodes due to loss of planned trajectories, 1 electrode trajectory was replanned in the operation theatre due to trajectory conflict with head-fixation equipment, 3 electrodes were omitted from implantation during the procedure after failure to attach the guiding screw due to insufficient bone thickness in a child.

#### Accuracy measurements

Postoperative CT/O-arm scan was used to segment and register individual electrode contacts models with pre-implantation imaging containing planned trajectories on the Stealth Station System (Medtronic Inc., USA). The accuracy measurements were performed manually by measuring lateral deviation in the plane perpendicular to the planned trajectory between the preoperative plan and the middle of the segmented contact. For target deviation, the distance between the tip of the planned trajectory and the center of segmented electrode was measured. For entry deviation, the distance between the trajectory at the entry to the grey matter and the center of segmented electrode was measured.

#### Study groups

The implantations were grouped according to implantation technique: G1 – surface registration and Vertek probe for trajectory alignment, G2 – rigid registration with conventional CT and Vertek probe, G3 – rigid registration with intraoperative O-arm CT and Vertek probe, G4 – rigid registration (conventional or intraoperative O-arm CT) and Autoguide system for trajectory alignment, G5 – rigid registration with intraoperative O-arm CT performed after sterile draping and Autoguide system. The study groups for precision analysis are described in Table 2.

**Table 2:** Description of study groups

		Study groups					
		G1	G2	G3	G4	G5	All
Implantations, n		5	8	6	8	9	**36**
Children / Adults		1 / 4	2 / 6	3 / 3	3 / 5	2 / 6	**11 / 25**
Male / Female		2 / 3	3 / 5	4 / 2	4 / 4	5 / 4	**18 / 18**
Age, years	Mean (SD)	26 (16)	28 (11)	21 (6)	21 (9)	25 (13)	**24 (11)**
	Median	18	27.5	20.5	21	23	**22.5**
	[Min – Max]	[8– 45]	[13–44]	[14–30]	[8–41]	[5–46]	**[5–46]**
No of electrodes per group, n		53	101	76	98	91	**419**
Implanted electrodes per patient [Min – Max]		[9–14]	[9–14]	[11–14]	[10–15]	[9–11]	**[9–15]**
Side of implantation Left / Right		35 / 18	61 / 40	25 / 51	98 / 0	40 / 51	**259 / 160**
Site of implantation Temporal / Other		1 / 52	17 / 84	18 / 58	19 / 79	13 / 78	**68 / 351**

G1 – surface registration and Vertek probe, G2 – rigid conventional CT registration and Vertek probe, G3 – rigid O-arm CT registration and Vertek probe, G4 – rigid conventional CT or O-arm CT registration and Autoguide robot, G5 – rigid sterile O-arm CT registration and Autoguide robot

### Data analysis

We analysed the accuracy of implantation using random effects linear modelling, treating the implantation technique group (G1 to G5) as a fixed effect and individual implantation as a random effect to account for electrode clustering within patients. Log-transformed error values were used to address the right-skewed distributions of entry and target errors. Separate models were fit for entry and target errors. The accuracy is summarized for each implantation technique as median error (in millimetres) and percentage change in median error for entry and target in a stepwise manner corresponding evolution of implantation technique. Categorical secondary outcomes (complications and SEEG result) summarized as percentages grouped by age. Data entry and descriptive analysis performed in Microsoft Excel (Microsoft Co., USA). Modelling performed with statsmodels 0.14.1 Python module using mixed linear models [38].The G1 group was used as reference for the linear model.

## Results

### Primary outcomes – Implantation accuracy

**Fig. 1:**
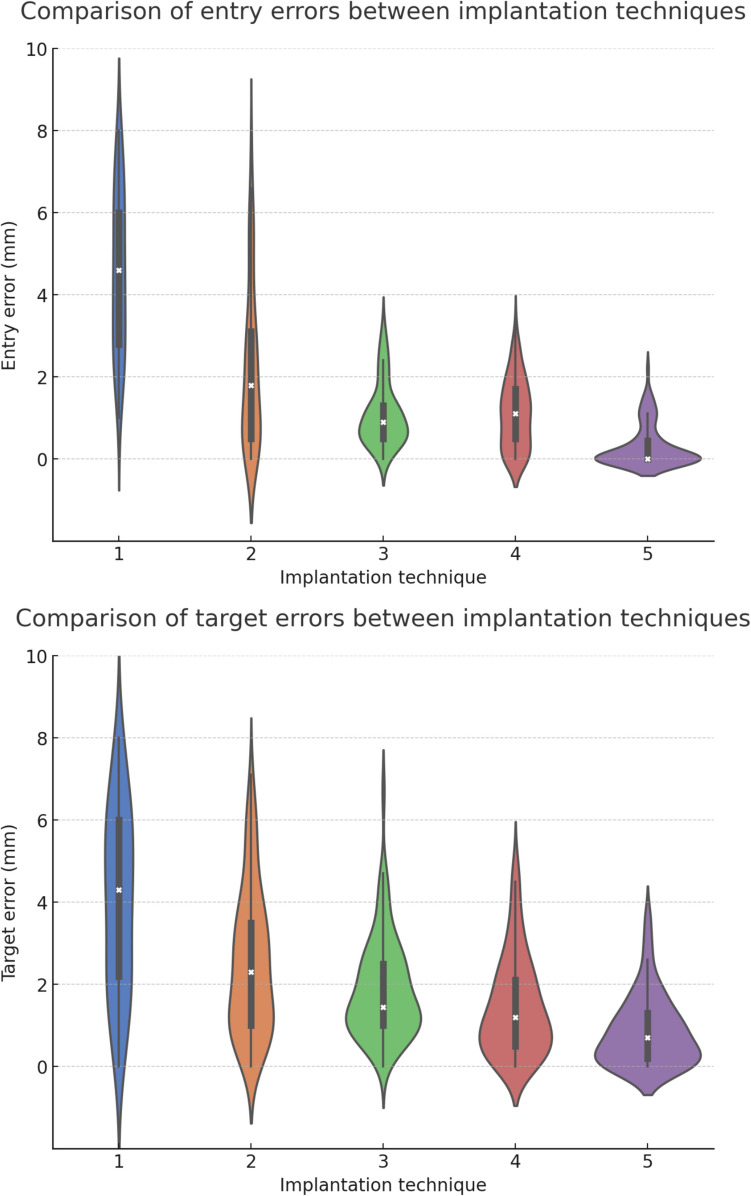
Distributions of the entry and target errors for the 5 different implantation techniques. 1 – surface registration and Vertek probe, 2 – rigid conventional CT registration and Vertek probe, 3 – rigid O-arm CT registration and Vertek probe, 4 – rigid conventional CT or O-arm CT registration and Autoguide robot, 5 – rigid sterile O-arm CT registration and Autoguide robot

The accuracy data is summarized in Table 3. Both entry and target error decreased with evolution in implantation technique. Overall, the resulting method improved accuracy by total of 72% (p < 0.001) and 51% (p < 0.001) for entry and target respectively. The distributions of entry and target error for different implantation methods are shown in Fig. 1.

**Table 3:** Implantation accuracy in study groups where relative improvement derived from linear mixed effects model with study group as a fixed effect and individual implantation as a random effect on log-transformed entry and target errors compared to the reference group

		Study groups				
		G1*	G2	G3	G4	G5
Entry error	Median, mm	4.6	1.8	0.9	1.1	0
	[Min – Max]	[1.0 – 8.0]	[0 – 7.7]	[0 – 3.3]	[0 – 3.3]	[0 – 2.2]
	Improvement, % (p)	-	38 (0.004)	47 (< 0.001)	50 (< 0.001)	72 (< 0.001)
Target error	Median, mm	4.3	2.3	1.45	1.2	0.7
	[Min – Max]	[0 – 8.0]	[0 – 7.1]	[0 – 6.7]	[0 – 5.0]	[0 – 3.7]
	Improvement, % (p)	-	17 (0.17)	22 (0.07)	35 (0.001)	51 (< 0.001)

G1* – surface registration and Vertek probe, G2 – rigid conventional CT registration and Vertek probe, G3 – rigid O-arm CT registration and Vertek probe, G4 – rigid conventional CT or O-arm CT registration and Autoguide robot, G5 – rigid sterile O-arm CT registration and Autoguide robot

### Secondary outcomes – Complications

There were no complications resulting in permanent deficit observed in our cohort. Among children, there were no bleeding or oedema. In one case, the attempt of insertion of a guiding screw in the temporal bone resulted in bone fracture and intracranial screw deviation due to poor bone quality after previous invasive investigation. The case resolved without sequelae.

Among adults we report 4 cases of bleeding: 1 epidural hematoma (G1), 1 small parenchymal bleed along electrode trajectory (G5), and 2 cases of profuse bleeding from skull bone on insertion of screw (G1 and G5). One case of moderate brain oedema (G1) and one case of severe post implantation headache (G1) were observed. One patient had a superficial skin infection treated with antibiotics. All cases were treated medically and resolved completely without need for further interventions.

10 electrodes *–* 7 (4.1%) in children and 3 (0.7%) in adults *–* were planned but not implanted due to insufficient bone quality/thickness at the entry point in 9 cases and conflict of trajectory with head clamp in 1.

Forty (75.5%) of all performed implantations resulted in actionable results: 32 (60.4%) patients were offered resective surgery, 3 (5.7%) Laser interstitial thermal therapy (LiTT), 2 patients (3.8%) achieved satisfactory seizure control after radiofrequency thermocoagulation (RF-TC) and no further interventions are planned. In 13 implantations (24.5%) no further intervention could be offered after first SEEG. Three (5.7%) patients were implanted twice among, them two were offered resective surgery as the result of second SEEG and in one case no further intervention could be offered. Secondary outcomes are summarised in Table 4.

**Table 4:** Clinical outcomes for SEEG investigations

		Clinical outcomes		
		Children (*n* = 16)	Adults (*n* = 37)	All (*n* = 53)
Complications, *n* (% of age group)	Bleeding	-	4 (10.8%)	**4 (7.5%)**
	Edema	-	1 (2.7%)	**1 (1.9%)**
	Infection	-	-	**-**
	Headache	-	1 (2.7%)	**1 (1.9%)**
	Bone fracture at screw insertion	1 (6.3%)	-	**1 (1.9%)**
SEEG outcome, *n* (% of age group)	Surgery	12 (75.0%)	20 (54.1%)	**32 (60.4%)**
	LiTT	1 (6.25%)	2 (5.4%)	**3 (5.7%)**
	RF-TC only	-	2 (5.4%)	**2 (3.8%)**
	New SEEG	1 (6.25%)	2 (5.4%)	**3 (5.7%)**
	No further interventions	2 (12.5%)	11 (29.7%)	**13 (24.5%)**
Total implanted electrodes, *n*		169	410	**579**
Suboptimal investigation, *n* (% of total electrodes per age group)	No. of planned but not implanted electrodes	7 (4.1%)	3 (0.7%)	**10 (1.7%)**
	Electrode detachment during SEEG recording	13 (7.7%)	2 (0.5%)	**15 (2.6%)**

*LiTT *Laser interstitial thermal therapy, *RF-TC *radiofrequency thermocoagulation

## Discussion

### Development of stereotactic method

The field of stereotactic neurosurgery, and its application in SEEG, has undergone significant evolution since its inception. The original Talairach method was successively supplanted, first, by frame-based systems, such as Leksell and Cosman-Roberts-Wells frames, which are now being increasingly replaced by frameless robot-assisted or fully robotic techniques[21]. The innovation continues in large epilepsy surgery centers, in terms of planning, image registration, head fixation, trajectory alignment, skull and dura penetration, and electrode placement methods [6, 10, 11, 46, 47]. Advances in image-guided surgery systems, computer-aided planning, manual and robotic trajectory alignment devices contributed to overall improvement of precision, safety, and consistency of SEEG implantations using frameless, manual or robotic systems. Such systems invariably improve efficiency of the procedures [15, 26, 40]. The accuracy of these novel systems varies across different methods and practicing centers, and are not in general superior to frame-based approaches [13, 19, 22, 35, 42].

### Implantation accuracy

The current study shows that high implantation accuracy can be achieved with frameless navigation using rigid registration under sterile conditions, to prevent movement of the navigation antenna from draping the patient. This is important knowledge for centers involved in SEEG where stereotactic robots are not available.

The final implantation method resulted in a median target error of 0.7 mm and a median entry error of 0 mm, which is comparable to implantations using dedicated stereotactic robot from a single center study where median entry error was 0.8 mm and median target error 1.6 mm [5]. In a recent meta-analysis of robotic SEEG implantations, comparing four different robots, mean entry error was 1.48 mm and mean target error 2.13 mm with only minor differences between the devices [43].

In our stepwise work on improving SEEG implantation precision, we have focused on registration and trajectory alignment techniques. For registration, we introduced rigid conventional then rigid O-arm based and finally rigid O-arm based sterile registration with the patient draped, and this study clearly shows how this improves accuracy, and also is consistent with previous findings, where rigid registration is a key factor in reducing deviations [41].

To improve trajectory alignment, we introduced the Autoguide, where alignment can be adjusted and corrected, compared to the Vertek probe. The effect of this on accuracy was not significant. One study has shown robotic SEEG to be significantly more accurate than frameless techniques, however this study did not use rigid registration for the frameless SEEG [37]. Overall, the evidence that stereotactic robots increase SEEG implantation accuracy compared to stereotactic frames is contradicting, and if there is an effect it is weak [14, 32, 40, 41, 43, 48]. In our experience the robot reduces operator-dependent variability, facilitates complex electrode insertions and decreases implantation time, and the latter was also found in a recent review [43]. We introduced sterile registration into our workflow to minimize the risk of moving the neuronavigation antenna when draping the patient and when changing from a non-sterile to a sterile antenna. To our knowledge, the effect of sterile registration using the O-arm on precision in frameless navigation has not been previously studied. The effect of this was small, but it seems to further reduce the maximum and median entry error.

### Clinical outcomes

The most severe complication in SEEG is intracranial hemorrhage, which occurred in 7.5% of patients. All hemorrhages were asymptomatic and identified on postoperative imaging only, which is in line with reported incidence of asymptomatic bleeds along the electrode shaft from 2.8% to 12.1% [15, 44]. In a meta-analysis of SEEG procedures in over 2500 patients, the incidence of complications was 1.3%, with 0.6% permanent neurological deficit and 0.3% mortality [30]. The total complication rates observed in this study are higher, as we also included asymptomatic bleedings and serious headaches, but in line with the study above, we had no permanent deficits.

Pediatric SEEG has special considerations due to bone thickness but can be employed with sufficient accuracy even in children with a bone thickness of 2 mm [28, 29, 33, 39]. Some studies demonstrate lower precision of electrode placement and higher complication rate in younger patients, especially with preceding bone defects [37, 45]. We did observe a higher degree of sub-optimal investigations in children (11.8%) versus adults (1.2%) due to electrode omissions during operation related to bone quality (4.1% in children vs 0.7% in adults) and detachment of guiding screws during recordings (7.7% in children vs 0.5% in adults). This did not affect the result of the investigation in terms of further therapeutic intervention, nor did it result in any permanent morbidity, similarly to data from larger pediatric series using SEEG [1, 9]. SEEG investigations resulted in a treatment (resection, laser ablation or radiofrequency thermocoagulation) in 75.5% of patients (87.5% in children, 70.3% in adults). This is comparable to data from high-volume centers where 67–80% of patients had a surgical procedure after SEEG [1, 7, 36].

### Limitations

The small sample size and single-centre design limit the generalizability of these findings. Moreover, accuracy measurements were unblinded, potentially introducing bias into the data. Exclusion of outliers, due to known methodological failures, limits the understanding of the system’s sensitivity to external factors such as movement of the navigation antenna. Retrospective design and limited number of patients per group precludes independent evaluation of underlying learning curve bias towards better accuracy measures in the most recent cases.

The lateral deviation method employed for accuracy measurements did not account for depth-related errors, which could underestimate total implantation error. Some previous studies employed Euclidean distance as a more comprehensive method for precision assessment [5, 17]. Although measuring only lateral deviation may obscure issues such as electrodes being implanted too deeply or too shallowly, affecting clinical SEEG outcomes, the safety of implantation is mostly affected by accuracy of lateral deviation, which is used to determine the safety margin to surface and deep vessels. Other factors, independent of implantation technique, such as angle of approach, stylet technique, electrode bending, and tissue properties along the planned trajectory, can influence implantation accuracy due to deviations at transitions from CSF to grey matter or shifts along the sulcal patterns; however, we didn´t specifically account for those factors in this study[16, 18, 23, 25, 27, 34].

## Conclusion

This study demonstrates that sub-millimeter precision in SEEG implantation can be achieved without frame or expensive specialised robots using a standard neuronavigation system and intraoperative sterile registration. Key factors for high precision are rigid and sterile registration, which minimizes the median error and range for target and entry points in placement of SEEG electrodes. The method is safe, accurate and is applicable in all age groups.

## Data Availability

Data is available in the manuscript and additional data can be accessed from the corresponding author.
